# Needle Cricothyrotomy Connection Options Provide Similar Oxygen Flow Rates

**DOI:** 10.7759/cureus.100277

**Published:** 2025-12-28

**Authors:** Natalie Oberhauser-Lim, Elioenai A Morales, Austin G Rotinsulu, Angela N Torres, Timothy P Young

**Affiliations:** 1 Pediatric Emergency Medicine, Loma Linda University School of Medicine, Loma Linda, USA; 2 Pediatric Emergency Medicine, Loma Linda University Medical Center, Loma Linda, USA; 3 Emergency Medicine, Loma Linda University School of Medicine, Loma Linda, USA

**Keywords:** emergency airway, needle cricothyrotomy, oxygenation, pediatric airway, resuscitation

## Abstract

Needle cricothyrotomy is an emergency airway procedure during which a large-bore intravenous catheter is inserted through the cricothyroid membrane into the trachea. One challenge of this procedure is connecting the intravenous catheter to the oxygen source. Multiple versions of catheter-to-oxygen connections have been described in the literature. We set out to compare the flow of oxygen through these setups to determine if any of the setups were superior to others. We measured the flow out of the intravenous catheter with an oxygen flow meter connected via a 3D-printed adaptor piece. Flow rates were the same through all setups. However, during our experiment, we identified advantages and disadvantages related to the connection configurations, which can be used to determine the best option for the clinical scenario.

## Introduction

Needle cricothyrotomy is the emergency airway procedure of choice for infants and young children in a “can’t intubate, can’t oxygenate” scenario. In infants, the cricothyroid membrane is too small to accommodate the endotracheal tube (ETT) or tracheostomy tube that would be used in an open cricothyrotomy [1,2]. In young children, the cricothyroid membrane may be large enough for a small ETT, but insertion carries a higher risk of damage to surrounding structures such as the thyroid and cricoid cartilage [3,4]. A needle cricothyrotomy can be performed using a commercially available kit or less specialized equipment that is more readily available. In the latter scenario, a large-bore intravenous (IV) catheter is inserted into the cricothyroid membrane. The catheter is then advanced into the upper airway. Various sizes have been recommended, ranging from 10 to 18 gauge catheters [3-5]. Prior experiments have described air flow rates through IV catheters of different sizes, with larger catheters permitting higher flow [6]. We teach our learners to use a 14-gauge catheter, as these are the largest diameter catheters stocked in our department. This size is also recommended by Pediatric Advanced Life Support guidelines [7] and within the recommended range of sizes [3-5].

The preferred oxygen route for younger children is typically a bag valve mask (BVM) connected to 100% oxygen [3,5]. This is also a good option for older children when a jet ventilator is not available. However, there is no way to directly attach a BVM to an IV catheter.

Multiple options for connecting the IV catheter to the oxygen source have been described in the literature [4,8], but it was unclear if the different connections would affect oxygen flow through the inserted IV catheter. We hypothesized that some of the longer connections may cause increased resistance and decreased flow. We compared the flow through these connection options to determine any appreciable difference in resultant flow out of the IV catheter.

## Technical report

**Figure 1:**
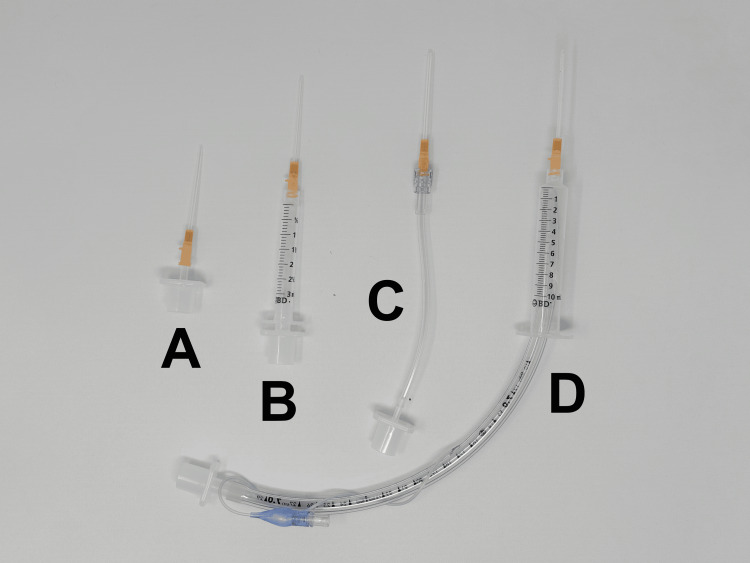
Four options for connecting an IV catheter to a bag valve mask A) a 3.0 ETT adapter connected directly to the catheter, B) a 7.0 ETT adapter connected to a 3 mL plungerless syringe, C) a 2.5 ETT connected to a cut IV tubing set, and D) a 7.0 ETT inflated in a 10 mL plungerless syringe ETT: endotracheal tube

We designed a simple experiment to compare flow rates between four described needle cricothyrotomy connection set ups: A) a 3.0 ETT adapter, B) the combination of a 7.0 ETT adapter and a 3 mL syringe without the plunger, C) a 2.5 ETT adapter inserted into a cut portion of IV extension tubing, and D) a 7.0 ETT with the cuff inflated in a 10 mL syringe without the plunger (Figure 1) [4,8].

**Figure 2:**
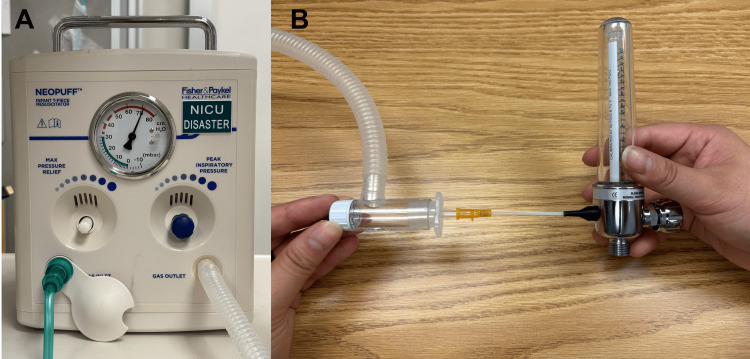
NeoPuff machine (image A) connected to the 3.0 endotracheal tube option, attached to an oxygen flow meter by way of a custom 3D-printed plastic connector (image B)

We connected the tip of the IV catheter to the flow meter via a custom 3D-printed polyethylene terephthalate glycol piece. Our 3D-printed connector can be downloaded by clicking on this link: https://tinyurl.com/flowmeterconnector. For consistency, during our measurements, we used the same oxygen source, IV catheter, 3D-printed adapter, and flow meter. The only portion that was changed was the connector between the IV catheter and the oxygen source. Therefore, any potential air leak in the circuit should have been consistent between setups. We measured flow from the end of the IV catheter with an oxygen flow meter (Figure 2).

We considered using a BVM to deliver the flow of oxygen, but felt that this would cause too much pressure variability. We considered a jet ventilator setup, but this is not readily available at our institution and is not recommended for children under five years old due to the risk of barotrauma [3]. Instead, we used a NeoPuff machine (Fisher and Paykel, East Tāmaki, Auckland, New Zealand) as our oxygen source to provide consistent pressures between the different circuits. The NeoPuff has the same connector as a BVM and provides adjustable pressure. There is a great degree of variability in recommendations for the amount of pressure and flow to deliver through needle cricothyrotomies in young children. We chose a pressure of 70 cm H2O, since this was the highest setting on the NeoPuff as well as the highest measurable pressure on our bag valve masks. Additionally, we wanted to use a pressure that would create a measurable flow on our oxygen flow meter. We also measured flow at 30 cm H_2_O to evaluate the connections at a lower pressure.

**Figure 3:**
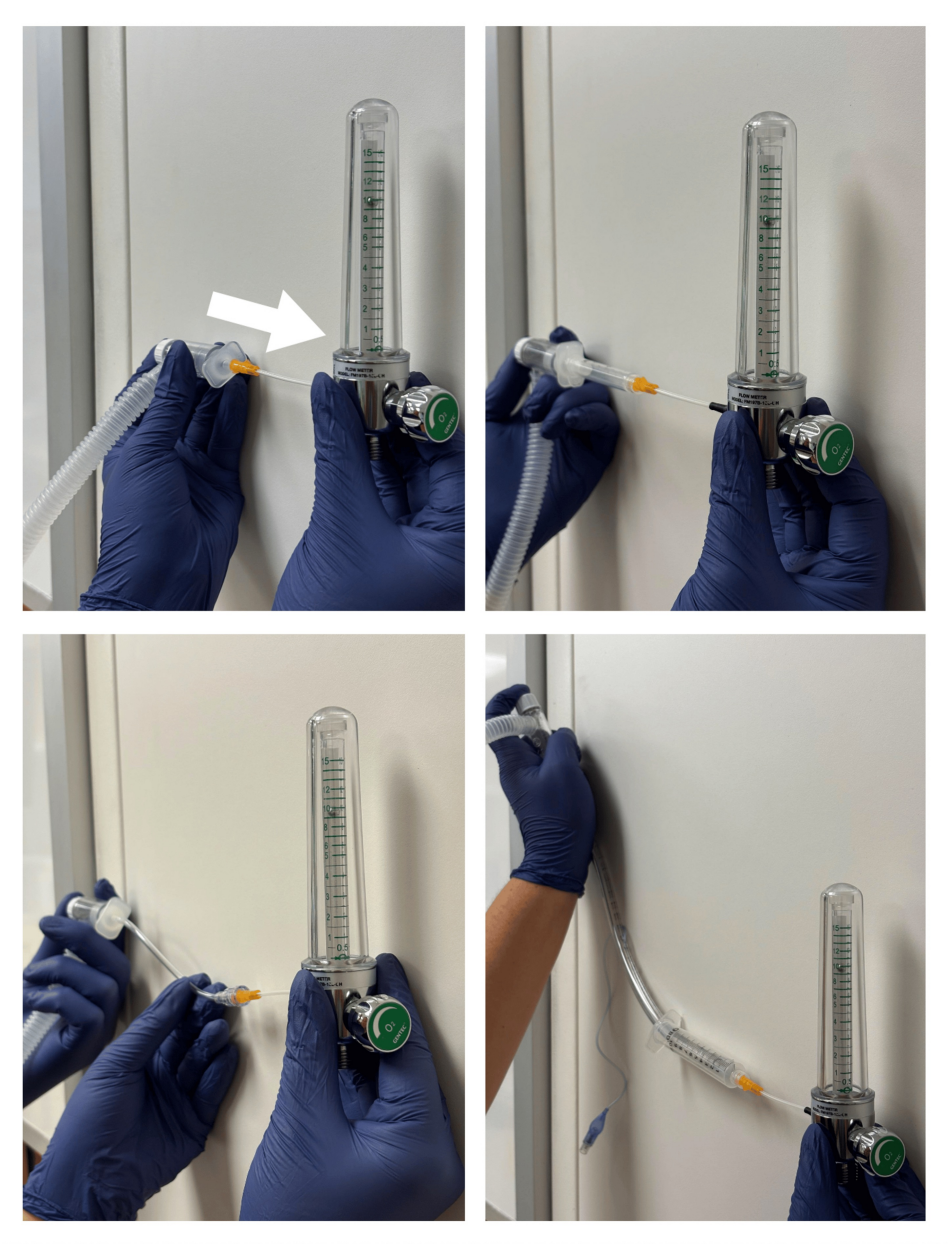
Flow was 10 L/min from all connection setups at an applied pressure of 70 cm H2O. The arrow shows the direction of oxygen flow.

We attached each of the four setups to the NeoPuff and set the flow of the NeoPuff to 70 cm H_2_O. When we initially turned on the air flow, the level of the stainless steel ball of the flow meter fluctuated as the pressure in the system stabilized. We allowed the ball to stabilize before obtaining a visual measurement. At 70 cm H_2_O, each of the four connection setups provided 10 L/min of air flow (Figure 3). At 30 cm H_2_O, each of the four connection setups provided 4.5 L/min of flow. We repeated these measurements by turning the circuit on and off five times, and visually, there was no difference in the measured flow on the oxygen flow meter.

## Discussion

We hypothesized that the length of IV tubing might increase resistance and impede flow or that the setup with the 7.0 ETT and 10 mL syringe might have leakage that would negatively impact flow; however, this was not the case. We found that at a pressure of 70 cm H_2_O, the NeoPuff generated approximately 10 L/min of flow through the end of the IV catheter in all setups. At a lower pressure of 30 cm H_2_O, the flow was also consistent between the different setups and produced approximately 4.5 L/min of flow.

We found that flow rates were similar in the four circuits we tested. Our findings can be explained by Poiseuille's law [9], which describes laminar flow through a cylindrical pipe of constant cross-sectional diameter.

Q=ΔPπr4/8ηL

Where Q is the flow rate, ΔP is the pressure difference, r is the radius of the tube, η is the fluid viscosity for oxygen, and L is the length of the tube. Changes in all variables affect flow by a power of 1 except for radius, which affects flow by a power of 4. Total resistance in the four circuits we tested would have been summative since the connectors were arranged in series as follows:

Rtotal​ = Rcatheter​ + Rconnector1 ​+ Rconnector2​ + …

Since the resistance in the IV catheter was so much higher than other sections of the circuit due to the “power of 4” effect of Poiseuille’s law, the resistance in the 14-gauge IV catheter determined the resistance in all circuits as follows:

Rtotal​ ≈ Rcatheter​

For example, although setup C used a length of IV tubing that was relatively small (3 mm) and closer in diameter to the 14-gauge IV catheter (1.6 mm) than other connectors we used, the additional resistance was still negligible compared with the resistance through the 14-gauge IV catheter. The IV catheter acted as the bottleneck/rate limiter in each circuit.

In conducting this experiment, we encountered several practical advantages and disadvantages for each setup (Table 1). Because flow was functionally the same through all circuits, a clinician can use these factors when choosing a setup.

**Table 1:** Practical comparison of needle cricothyrotomy setups

Connector Set Up	Advantages	Disadvantages
3.0 ETT Adapter	Only requires a single piece for connection	High risk of kinking catheter; have to disconnect adapter from ETT 3.0 ETT, may not be easily accessible in all EDs
7.0 ETT Adapter and 3mL syringe	Uses common ED equipment	High risk of kinking the IV catheter; have to disconnect the adapter from the ETT
7.0 ETT and 10mL syringe	Uses common ED equipment. Does not require the disassembly of the ETT	Could come apart if the balloon is not adequately inflated
2.5 ETT adapter and IV tubing	Flexibility of the ETT decreases the risk of kinking the catheter	Have to disconnect the adapter from the ETT and cut the IV tubing 2.5 ETT, which may not be easily accessible in all EDs

Our experiment has several limitations. We used continuous rather than intermittent flow. Although intermittent flow better simulates ventilation, continuous flow allowed us to compare flow rates with less variability. We chose a pressure of 70 cm H2O for practical reasons, but acknowledge that it is lower than some recommendations and does not represent the maximum possible flow from an IV catheter. We did not measure precise flow rates; instead, we relied on visual measurements. Because flow rates were so similar, and keeping Poiseuille's law in mind, it is unlikely that there was any practical, meaningful difference between them. Lastly, there are commercial percutaneous translaryngeal ventilation kits available, so institutions with these kits may not need to assemble any of the connections we tested. However, in institutions like ours that do not have these premade kits, it is useful to be familiar with the various setups for needle cricothyrotomy.

## Conclusions

We found that oxygen flow rates were similar through the four connection setups that we tested. These findings are in accordance with Poiseuille’s law, which dictates that the flow rate is most impacted by the radius of a cylindrical pipe. Thus, the determining factor of the flow rate was ultimately the 14-gauge catheter, with minimal impact from the various connection setups. Each connection has a distinct set of practical advantages and disadvantages, so a clinician’s choice of setup can be dictated by these considerations or equipment availability.
